# Supplementary material for: Transcriptomic and Metabolic Analyses Reveal the Mechanism of Ethylene Production in Stony Hard Peach Fruit during Cold Storage
Source: Int J Mol Sci. 2021 Oct 20;22(21):11308. doi: 10.3390/ijms222111308 (PMC8583708; doi:10.3390/ijms222111308)
Supplement: Supplementary file 1 [file ijms-22-11308-s001.zip › Figure S and Table S.pdf]

Table S1. Statistics of RNA-seq data of each stony hard (SH) peach fruit sample.

Sample name	UMI reads	UMI mapped (%)	Multiple mapped reads (%)	Uniquely mapped reads (%)	Read-1 (%)	Read-2 (%)	Reads mapped to positive strand (%)	Reads mapped to negative strand (%)	Non-splice reads (%)	Splice reads (%)
CK01	37755154	35640765 (94.4)	530921 (1.41)	35109844 (92.99)	17794631 (47.13)	17315213 (45.86)	17547441 (46.48)	17562403 (46.52)	22860521 (60.55)	12249323 (32.44)
CK02	37783908	35448660 (93.82)	562739 (1.49)	34885921 (92.33)	17708330 (46.87)	17177591 (45.46)	17433129 (46.14)	17452792 (46.19)	22608838 (59.84)	12277083 (32.49)
CK03	38607372	36403098 (94.29)	584832 (1.51)	35818266 (92.78)	18193429 (47.12)	17624837 (45.65)	17896288 (46.35)	17921978 (46.42)	23161617 (59.99)	12656649 (32.78)
L1_1	36487772	34488689 (94.52)	853392 (2.34)	33635297 (92.18)	17195105 (47.13)	16440192 (45.06)	16855203 (46.19)	16780094 (45.99)	22245787 (60.97)	11389510 (31.21)
L1_2	40880792	38765505 (94.83)	918994 (2.25)	37846511 (92.58)	19210092 (46.99)	18636419 (45.59)	18942646 (46.34)	18903865 (46.24)	25082131 (61.35)	12764380 (31.22)
L1_3	36690964	34924120 (95.18)	816762 (2.23)	34107358 (92.96)	17334111 (47.24)	16773247 (45.71)	17082077 (46.56)	17025281 (46.4)	22842062 (62.26)	11265296 (30.7)
L2_1	37078806	35169332 (94.85)	679549 (1.83)	34489783 (93.02)	17618591 (47.52)	16871192 (45.5)	17266397 (46.57)	17223386 (46.45)	23342994 (62.96)	11146789 (30.06)
L2_2	38083194	36175273 (94.99)	753587 (1.98)	35421686 (93.01)	17979610 (47.21)	17442076 (45.8)	17738625 (46.58)	17683061 (46.43)	23719332 (62.28)	11702354 (30.73)
L2_3	34591170	32920334 (95.17)	694369 (2.01)	32225965 (93.16)	16331918 (47.21)	15894047 (45.95)	16129240 (46.63)	16096725 (46.53)	21428874 (61.95)	10797091 (31.21)

Abbreviations used are as follows: CK0, CP9 stored at room temperature for 0 days; L1, CP9 stored at low temperature 4 °C for 20 days; L2, CP9 stored at 4 °C for 40 days

Table S2. Quality of reads obtained by RNA-seq analysis of SH peach fruit samples.

Sample name	No. of raw reads	No. of clean reads	UMI reads	Raw data (Gb)	Clean data (Gb)	Error rate (%)	Q20 (%)	Q30 (%)
CK01	46988078	41103892	37755154	7.05	6.17	0.03	98.08	94.07
CK02	47547386	41226354	37783908	7.13	6.18	0.03	97.85	93.64
CK03	47726198	42112606	38607372	7.16	6.32	0.03	97.74	93.37
L1_1	49551870	39780928	36487772	7.43	5.97	0.03	97.93	93.77
L1_2	52280466	44535718	40880792	7.84	6.68	0.03	97.95	93.8
L1_3	47034346	39930250	36690964	7.06	5.99	0.03	97.98	93.89
L2_1	49577982	40482350	37078806	7.44	6.07	0.03	97.98	93.84
L2_2	48528418	41493836	38083194	7.28	6.22	0.03	98	93.98
L2_3	43443546	37708476	34591170	6.52	5.66	0.03	98.1	94.13

Table S3. Correlation matrix of RNA-seq samples.

**Sample**	**CK01**	**CK02**	**CK03**	**L1_1**	**L1_2**	**L1_3**	**L2_1**	**L2_2**	**L2_3**
CK01	1	0.994	0.994	0.752	0.754	0.751	0.74	0.747	0.747
CK02	0.994	1	0.994	0.753	0.754	0.751	0.74	0.747	0.747
CK03	0.994	0.994	1	0.752	0.753	0.749	0.737	0.745	0.747
L1_1	0.752	0.753	0.752	1	0.993	0.989	0.913	0.919	0.92
L1_2	0.754	0.754	0.753	0.993	1	0.992	0.916	0.921	0.921
L1_3	0.751	0.751	0.749	0.989	0.992	1	0.92	0.92	0.916
L2_1	0.74	0.74	0.737	0.913	0.916	0.92	1	0.99	0.983
L2_2	0.747	0.747	0.745	0.919	0.921	0.92	0.99	1	0.992
L2_3	0.747	0.747	0.747	0.92	0.921	0.916	0.983	0.992	1
CK21	0.953	0.953	0.954	0.759	0.76	0.756	0.75	0.758	0.76
CK22	0.953	0.954	0.953	0.759	0.761	0.758	0.752	0.759	0.759
CK23	0.954	0.954	0.954	0.76	0.761	0.758	0.751	0.758	0.758

Table S4. Expression profiles of genes related to hormone biosynthesis and signaling.

	Gene_ID	CK0	L1	L2
Ethylene	Prupe.6G235600 EIN2	61.5925703	58.24840057	27.8686312
	Prupe.1G034300 ETR2	13.00593977	143.5208565	311.3525208
	Prupe.2G176900 ACS1	0.633347231	47.69817504	160.8023851
	Prupe.3G209900 ACO1	4154.21	12665.78118	14640.68724
	Prupe.4G013800 ACO2	8.45395845	21.65807474	9.236840176
	Prupe.5G083500 ACS2	3.74260983	41.10120549	13.35549105
	Prupe.7G117700 CTR1	16.00762874	33.32787854	52.22840445
	Prupe.7G244300 EBF	12.82992998	17.43049736	48.20806285
	Prupe.6G018200 EIL3	29.23507649	59.40797295	67.56336783
IAA	Prupe.6G157500 YUC11	0.074608831	0.141273397	0.189238008
	Prupe.8G253300 TIR1	41.05531401	37.23140969	14.12823759
	Prupe.4G037200 TIR1	25.9610246	3.647098103	4.257593621
	Prupe.7G234800 AUX/IAA1	1.270731008	307.8730726	870.9916524
	Prupe.8G137900 GH3.1	5.375159287	92.85530699	314.8477489
ABA	Prupe.3G215000 MPK6	2.590487736	165.2632072	122.9372816
	Prupe.4G082000 NCED2	21.05746049	167.8907815	226.925148
	Prupe.4G150100 NCED3	2257.086656	246.7577221	474.5703715
	Prupe.5G013100 CYP707A	0.38228417	16.662729	9.042417296
	Prupe.7G133100 ZEP	300.3703113	1185.362694	144.2557406
	Prupe.2G256700 PYL	0.231353963	7.132851685	14.61476612
	Prupe.2G308200 PYL	92.88178132	90.99770281	144.3316977
	Prupe.5G036800 PYL	5.152704635	4.146400065	14.42569457
	Prupe.1G408100 PP2C	51.72482354	26.12293387	21.26089757
	Prupe.3G044900 PP2C	287.2251347	216.7184683	96.04647511
	Prupe.5G054500 SnRK2	82.60077718	184.8992203	194.0986687
	Prupe.7G138200 SnRK2	66.98009938	189.0648382	258.7269685
	Prupe.1G573300 SnRK2	55.93570074	129.9197846	137.9129948
JA	Prupe.5G035400 MYC2	21.09976308	38.54845102	56.27811422
	Prupe.8G174900 CHiB	2.138958313	15.3221552	73.13234643

Table S5. Expression profiles of genes related to cell wall metabolism.

Gene_id	CK0	L1	L2
Prupe.1G006800 PME	0	0.163366309	10.39322251
Prupe.1G529500 PME	0.014768333	15.17244934	56.37513503
Prupe.4G116600 PG	0.717756064	39.67015813	84.31721212
Prupe.5G047300 EXP	0.032829831	5.982546358	15.45326569
Prupe.5G057900 EXP	1.03892463	23.48306455	50.62989784
Prupe.6G101100 CAP	7.129074566	15.63895579	26.69541215
Prupe.6G195800 CAP	140.3205491	543.1113768	812.9640874
Prupe.1G262000 GAL	1.58232017	161.6766393	273.5831646
Prupe.6G099700 BGAL	5.469409588	89.64727553	178.0720849
Prupe.7G164600 F5H	0.159093847	0.188556315	11.12034222
Prupe.1G492800 BGAL	0.792066608	26.4464282	18.34534673
Prupe.1G525500 CSC	0.056000287	18.31585994	1.502365839

Table S6. Expression profiling of genes related to lipid metabolism during cold storage.

Gene_ID	CK0	L1	L2
Prupe.1G003300 KAT	156.3045772	907.4286647	857.6219912
Prupe.1G034900 DCI	12.10038531	343.3404459	440.7650997
Prupe.1G067900 PEPC	23.82798293	80.64749135	91.53341529
Prupe.1G130000 PLDδ	15.25771205	26.4354199	29.38505355
Prupe.1G214000 OBD	54.88222364	7.003515759	40.55456502
Prupe.1G220600 DCI	8.901501847	134.4426622	128.7109298
Prupe.1G228900 LDAP	24.7218244	0.049989904	0.110052782
Prupe.1G309100 PPDC	18.39211893	3.307794416	4.373096095
Prupe.1G317400 LPPα	49.36654941	47.81230914	49.56102927
Prupe.1G332000 CLO	19.48657311	1968.989202	3509.826609
Prupe.1G333400 LpxC	13.4079403	0.279150371	2.802567021
Prupe.1G345300 SDR	22.1243144	0.357116058	0.163169235
Prupe.1G363200 SYT	45.13078409	96.36614845	18.69641574
Prupe.1G401700 HD	28.08419593	4.960128564	4.010687069
Prupe.1G411100 Δ8SLD	18.60802771	2.334427016	1.931083184
Prupe.1G434100 OBL	0.065788292	13.56631824	41.00337891
Prupe.1G440100 PLA2-II	0	13.58846679	8.316299139
Prupe.1G584800 LCB	37.96626124	47.1541821	25.71731291
Prupe.2G098700 PLC	7.830767292	851.3655542	871.0214531
Prupe.2G152100 PLDδ	13.39048411	26.61696503	21.90494724

Gene_ID	CK0	L1	L2
Prupe.2G214300 PLA2-III	9.181108816	37.11720043	40.17224985
Prupe.2G231700 DCI	17.91330285	78.91998862	80.6238108
Prupe.2G234700 ECR	18.85970478	2.836546098	5.577809002
Prupe.3G052000 PCP	2.696111497	8.099675411	26.90649748
Prupe.3G084800 PLDα	387.8060164	68.53333779	53.95720755
Prupe.3G091800 GK	45.96366243	9.948532304	7.357465669
Prupe.3G163400 LDAP	42.91865327	1.421140363	2.33888367
Prupe.3G207800 MPT	19.78319872	76.79301445	4.140992925
Prupe.3G219100 ISL	119.7435642	26.98918001	22.19653021
Prupe.3G228600 DGK	32.9787846	22.55968302	97.58996625
Prupe.3G285900 ALA	25.26213202	6.836157849	4.324046357
Prupe.4G011700 CDS	12.26224236	23.82094827	10.20750822
Prupe.4G015900 MCD	16.85992669	0.614238257	1.124373903
Prupe.4G078200 KASI	34.66192834	3.641023506	5.198590001
Prupe.5G075100 KAS	17.4601926	17.55291713	19.19556389
Prupe.5G080500 MTM	48.83952597	6.218953017	19.81449792
Prupe.5G214200 ALA	7.954917103	15.61163955	12.04816235
Prupe.5G216500 OSP	22.17223475	3.51407141	0.545020949
Prupe.6G015200 ENR	14.9444097	1.092933952	2.46931194
Prupe.6G056100 FAD	7.200285013	521.4209208	672.8138104
Prupe.6G171900 KSR	22.86679516	14.1823804	13.39917134

Gene_ID	CK0	L1	L2
Prupe.6G197600 KSR	30.85216098	23.27445835	25.53362982
Prupe.6G240200 LPPγ	21.34917107	7.674290387	3.050974627
Prupe.6G269200 NCER	15.5978565	0.525556263	2.696385845
Prupe.6G321100 LDAP	15.91365697	40.50091293	98.37850034
Prupe.6G335300 ACP	36.12351984	2.393624692	8.779635077
Prupe.6G364000 PLA	22.36681664	0.853021601	1.900226681
Prupe.6G365100 SAD	1408.360497	608.2735371	691.394314
Prupe.6G365200 SAD	53.16140707	22.05612847	23.51339749
Prupe.7G076200 ENR	17.92840302	0.230325841	0.611821912
Prupe.7G076500 FAD	134.0908888	4431.950277	7192.61683
Prupe.7G084800 LCB	3.129003085	16.70523733	6.556997515
Prupe.7G219900 GCS	186.1089708	42.79564044	418.8584909
Prupe.7G221100 ENR	880.1470854	154.9312882	91.60004401
Prupe.7G221100 ENR	880.1470854	154.9312882	91.60004401
Prupe.7G251400 MGD	8.449025277	11.73366249	34.72457199
Prupe.7G262100 AAPT	49.71142835	136.9744395	25.86408244
Prupe.8G018700 SAD	1.650302286	0	22.8926337
Prupe.8G045600 SBH	10.22904812	1.320787834	1.498047716
Prupe.8G096100 KAS	14.9376561	0.190124249	0.085097653
Prupe.8G261500 MGL	0.091970489	6.492187116	17.00934308
Prupe.8G270300 PLIP1	78.60939132	292.7540127	285.455773

Table S7. List of genes related to cell wall modification, lipid metabolism, and phytohormone biosynthesis in the co-expression network of Figure 7c.

Gene_ID	Label	Gene_ID	Label
Prupe.1G006800	PME	Prupe.5G057900	EXP
Prupe.1G034300	ETR2	Prupe.5G063900	PGPP
Prupe.1G130000	PLDδ	Prupe.5G083500	ACS2
Prupe.1G232400	LOX13	Prupe.6G018200	EIL3
Prupe.1G262000	GAL	Prupe.6G056100	FAD
Prupe.1G306100	AOC	Prupe.6G099700	GAL
Prupe.1G374600	SnRK2	Prupe.6G101100	CAP
Prupe.1G408100	PP2C	Prupe.6G195800	CAP
Prupe.1G411100	Δ8SLD	Prupe.6G235600	EIN2
Prupe.1G529500	PME	Prupe.6G364000	PLA
Prupe.1G560600	PSD	Prupe.7G076500	FAD
Prupe.1G573300	SnRK2	Prupe.7G084800	LCB
Prupe.2G058400	EIN3	Prupe.7G094500	DGD
Prupe.2G058500	EIN3	Prupe.7G117700	CTR1
Prupe.2G176900	ACS1	Prupe.7G133100	ZEP
Prupe.2G221600	PGPS	Prupe.7G138200	SnRK2
Prupe.2G256700	PYL4	Prupe.7G164600	F5H
Prupe.2G308200	PYL	Prupe.7G178500	CHiB
Prupe.3G044900	PP2C	Prupe.7G244300	EBF
Prupe.3G084800	PLDα	Prupe.8G018700	SAD
Prupe.3G209900	ACO1	Prupe.8G174900	CHiB

**Gene_ID**	**Label**	**Gene_ID**	**Label**
Prupe.3G228600	DGK	Prupe.2G098700	PLC
Prupe.3G239900	AOC	Prupe.1G305900	ERF
Prupe.3G311200	DGAT	Prupe.1G393000	WRKY
Prupe.4G011700	CDS	Prupe.8G234900	HSF
Prupe.4G013800	ACO2	Prupe.6G278600	Trihelix
Prupe.4G082000	NCED3	Prupe.1G037900	ERF
Prupe.4G116600	PG	Prupe.7G001400	NAC
Prupe.4G150100	NCED2	Prupe.2G256900	ERF
Prupe.5G035400	MYC2	Prupe.6G343100	bZIP
Prupe.5G036800	PYR1	Prupe.4G055600	ERF
Prupe.5G047300	EXP	Prupe.4G066400	WRKY
Prupe.5G054500	SnRK2	Prupe.3G252600	TCP

Table S8. List of genes related to cell wall modification, lipid metabolism, and phytohormone biosynthesis in the co-expression network of Figureure 7d.

Gene_id	Label
Prupe.1G525500	CSC
Prupe.2G316100	Cyt-b5
Prupe.3G025600	PLY1
Prupe.7G121600	XYL
Prupe.8G242600	TBL
Prupe.5G090000	CBF
Prupe.6G192200	SnRK2
Prupe.1G440100	PLA2-II
Prupe.1G003300	KAT
Prupe.3G215000	MP6K
Prupe.5G013100	ABH
Prupe.7G204800	PYL
Prupe.5G083500	ACS2
Prupe.2G205700	C2H2
Prupe.2G146600	G2-like
Prupe.3G027400	LOB

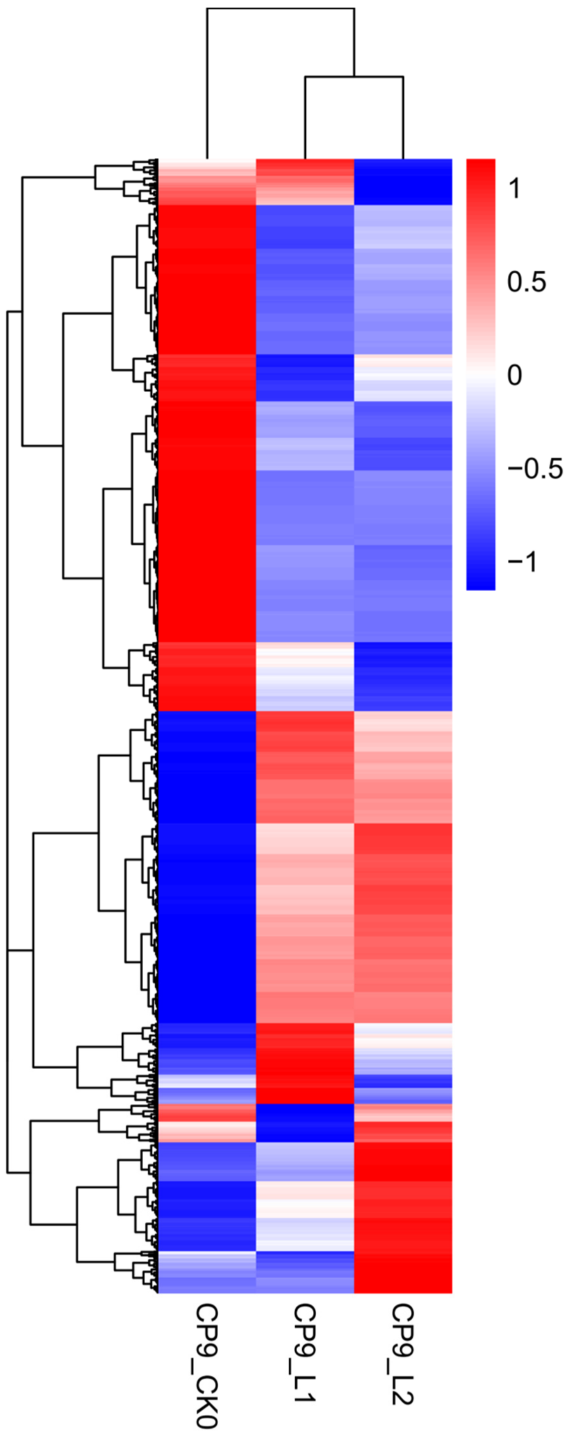

Figure S1. Hierarchical clustering analysis (HCA) of differentially expressed genes (DEGs) ($P < 0.05$; $|log2FC| \geq 2$). Colors in the heatmap indicate scaled expression level of genes (log2FPKM) across different samples. The color gradient ranging from blue to red represents low to high gene expression levels, respectively. Abbreviations used are as follows: CP9_CK0, CP9 stored at room temperature for 0 days; CP9_L1, CP9 stored at low temperature 4 °C for 20 days; CP9_L2, CP9 stored at 4 °C for 40 days.

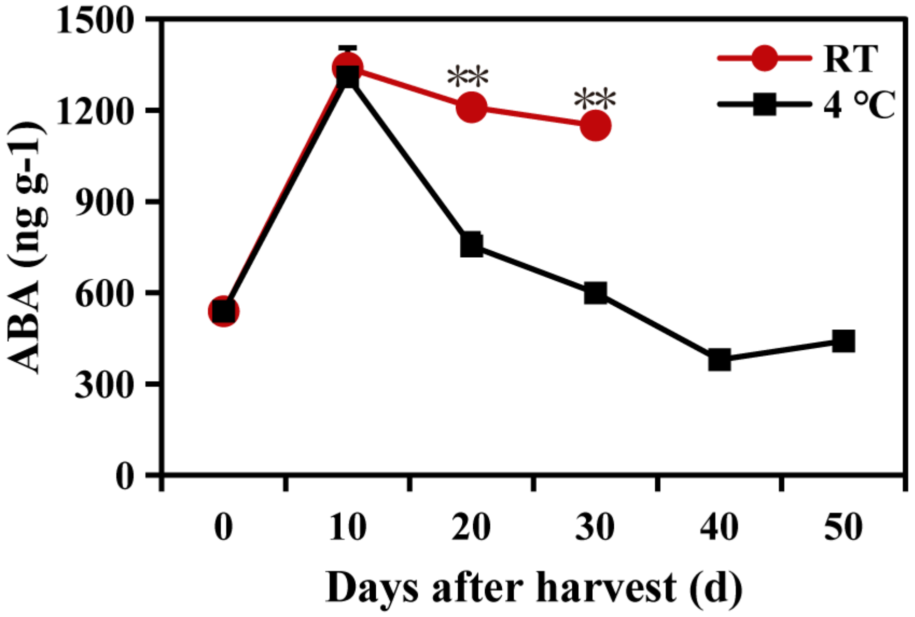

Figure S2. Changes in abscisic acid (ABA) content in stony hard (SH) peach fruit at harvest, during cold storage, and during subsequent ripening. Asterisks (**) indicate significant differences ($P < 0.01$; Student's t-test). Abbreviations used are as follows: CK0 (stored at room temperature for 0 d)；L1, (stored at 4 °C for 20 d); and L2, (stored at 4 °C for 40 d). Hormone content in the sample: (mg kg-1)=c*V/1000/m, where c is the concentration value obtained by substituting the integrated peak area ratio in the sample into the standard curve, V is the volume at redissolution, and m is the quality of the sample.

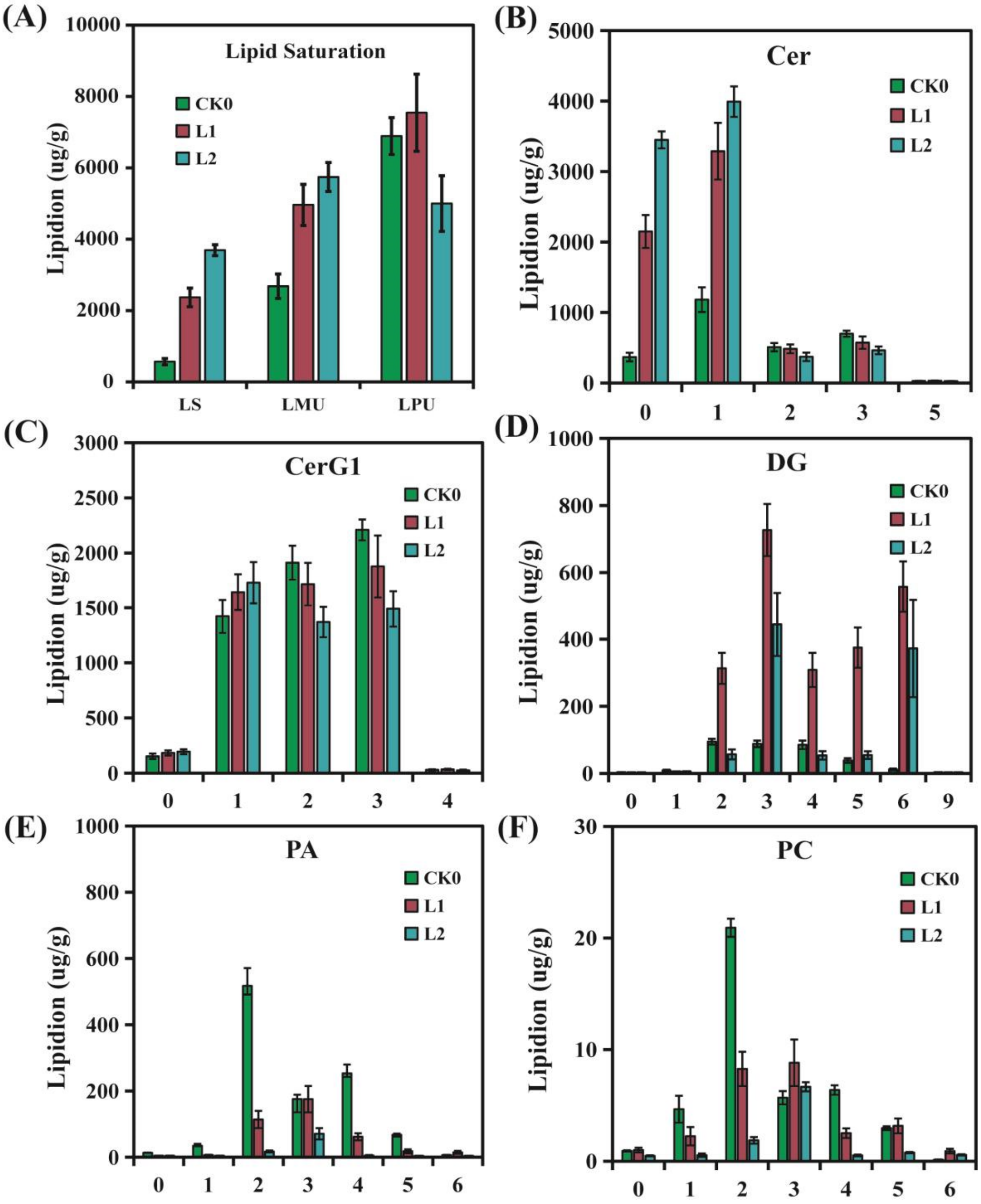

Figure S3. Lipid saturation analysis of SH peach fruit at the indicated time points during cold storage. Asterisks (**) indicate significant differences (P < 0.01; Student's t-test). Abbreviations used are as follows: LS, Lipid Saturation; LMU, Lipid monounsaturation; LPU, Lipid polyunsaturation; CK0, (stored at room temperature for 0 d); L1, (stored at 4 °C for 20 d); L2, (stored at 4 °C for 40 d).

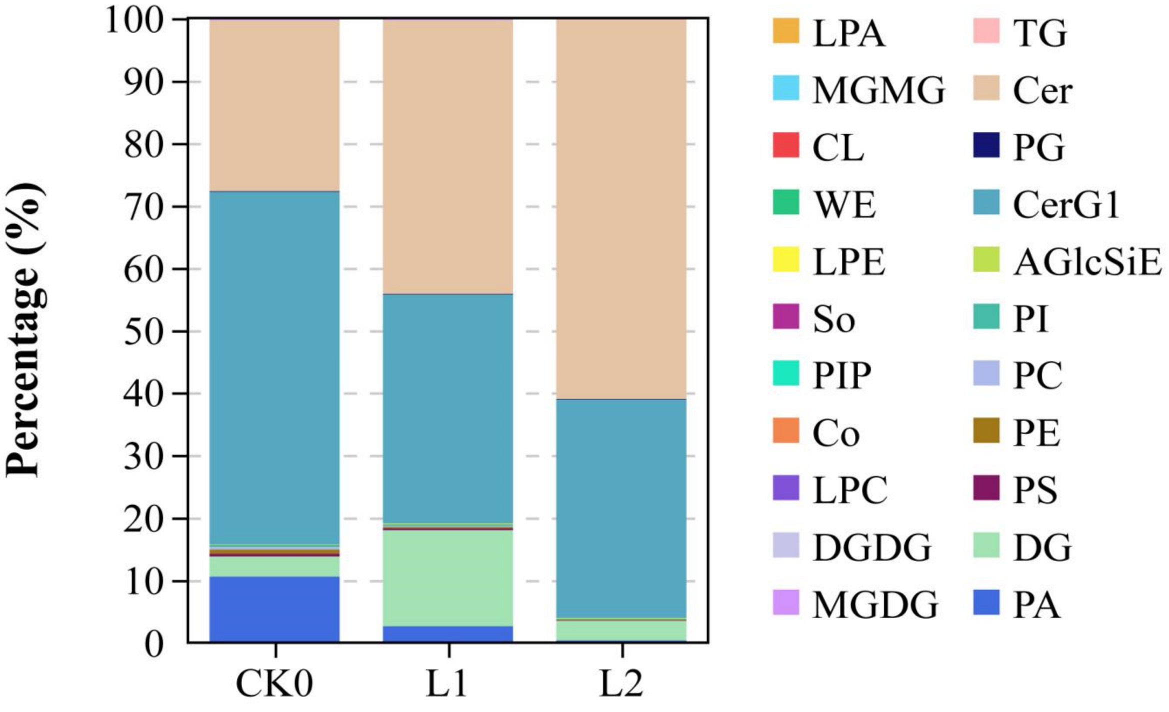

Figure S4. Changes in lipid composition of SH peach fruit during cold storage. Different colors represent different lipids. Abbreviations used are as follows: CK0, CP9 stored at room temperature for 0 days; L1, CP9 stored at low temperature (4 °C) for 20 days; L2, CP9 stored at 4 °C for 40 days.

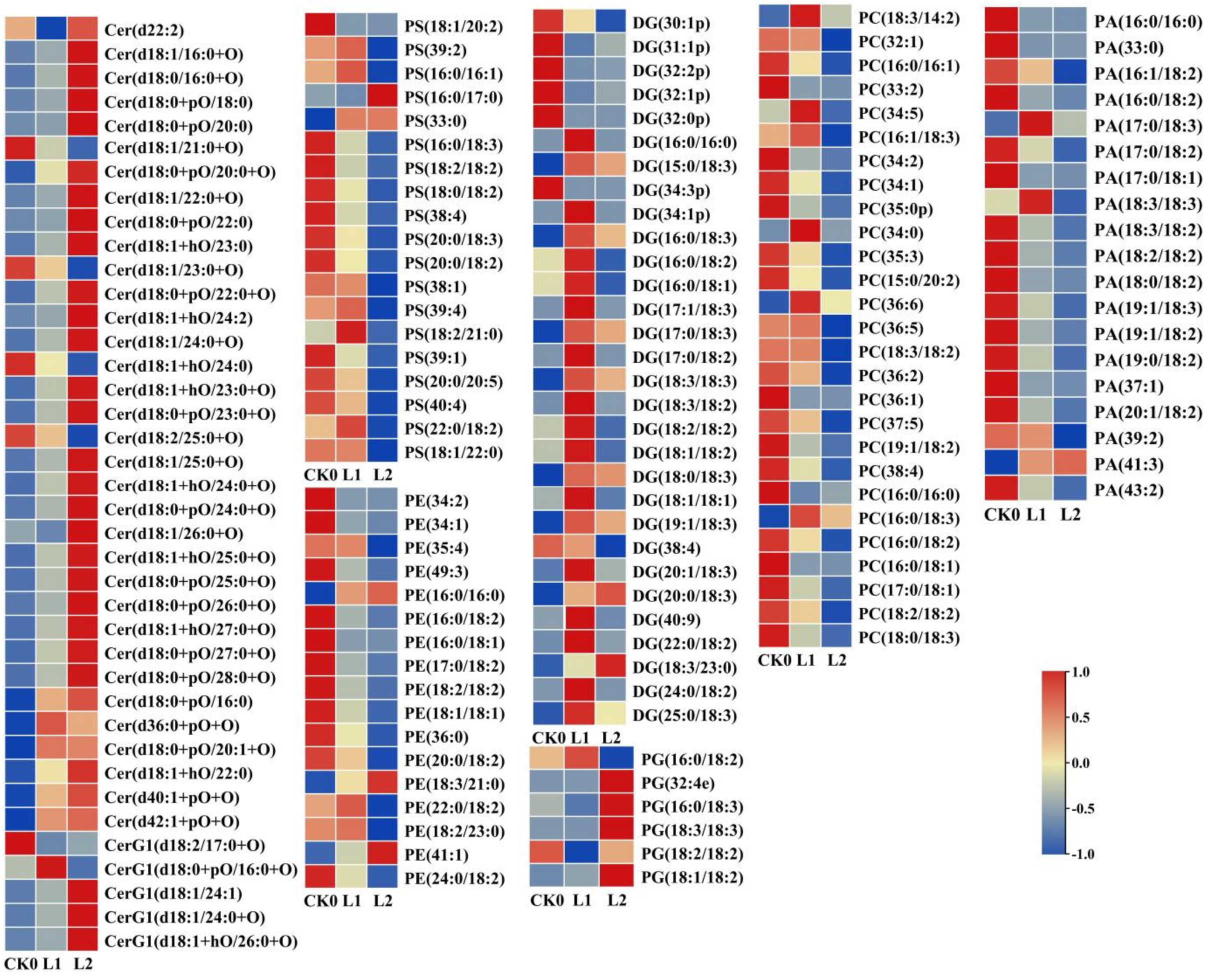

Figure S5. Heatmap of lipid contents showing differential accumulation. Blue, white, and red colors indicate low, medium, and high contents, respectively. Abbreviations used are as follows: Cer, ceramide; CerGl, monoglycosylceramide; DG, diacylglycerol; PA, phosphatidic acid; PC, phosphatidylcholine; PE, phosphatidylethanolamine; PS, phosphatidylserine; PI, phosphatidylinositol; Abbreviations used are as follows: CK0, CP9 stored at room temperature for 0 days; L1, CP9 stored at low temperature (4 °C) for 20 days; L2, CP9 stored at 4 °C for 40 days.

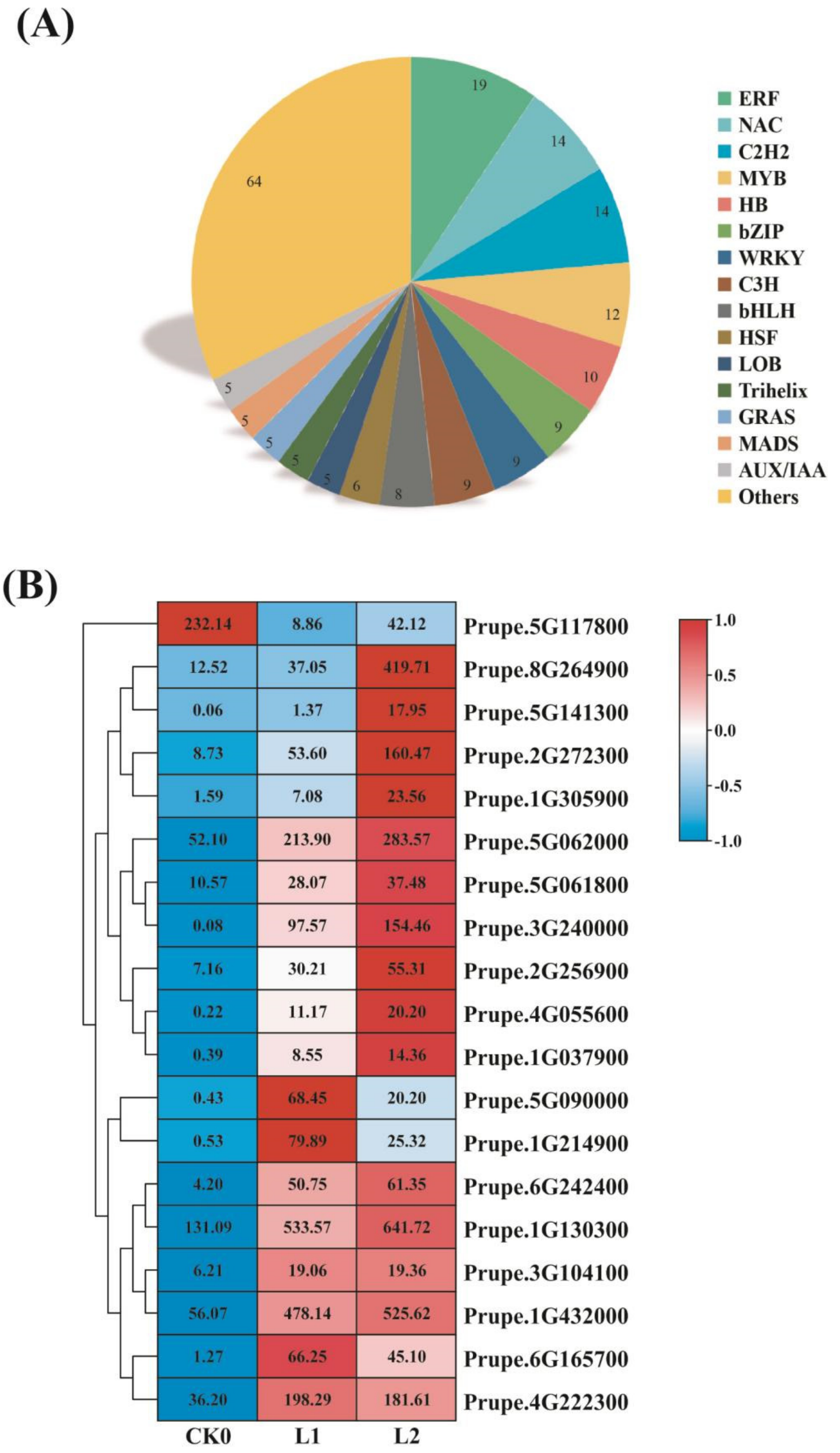

Figure S6. Transcription factor (TF) genes differentially expressed during cold storage: (A) Number of differentially expressed TF genes in different classes; and (B) Expression profiles of ERFs. Abbreviations used are as follows: CK0, CP9 stored at room temperature for 0 days; L1, CP9 stored at low temperature (4 °C) for 20 days; L2, CP9 stored at 4 °C for 40 days.
