# Supplementary figures and images for: Transcriptomic and Metabolic Analyses Reveal the Mechanism of Ethylene Production in Stony Hard Peach Fruit during Cold Storage
Source: Int J Mol Sci. 2021 Oct 20;22(21):11308. doi: 10.3390/ijms222111308 (PMC8583708; doi:10.3390/ijms222111308)

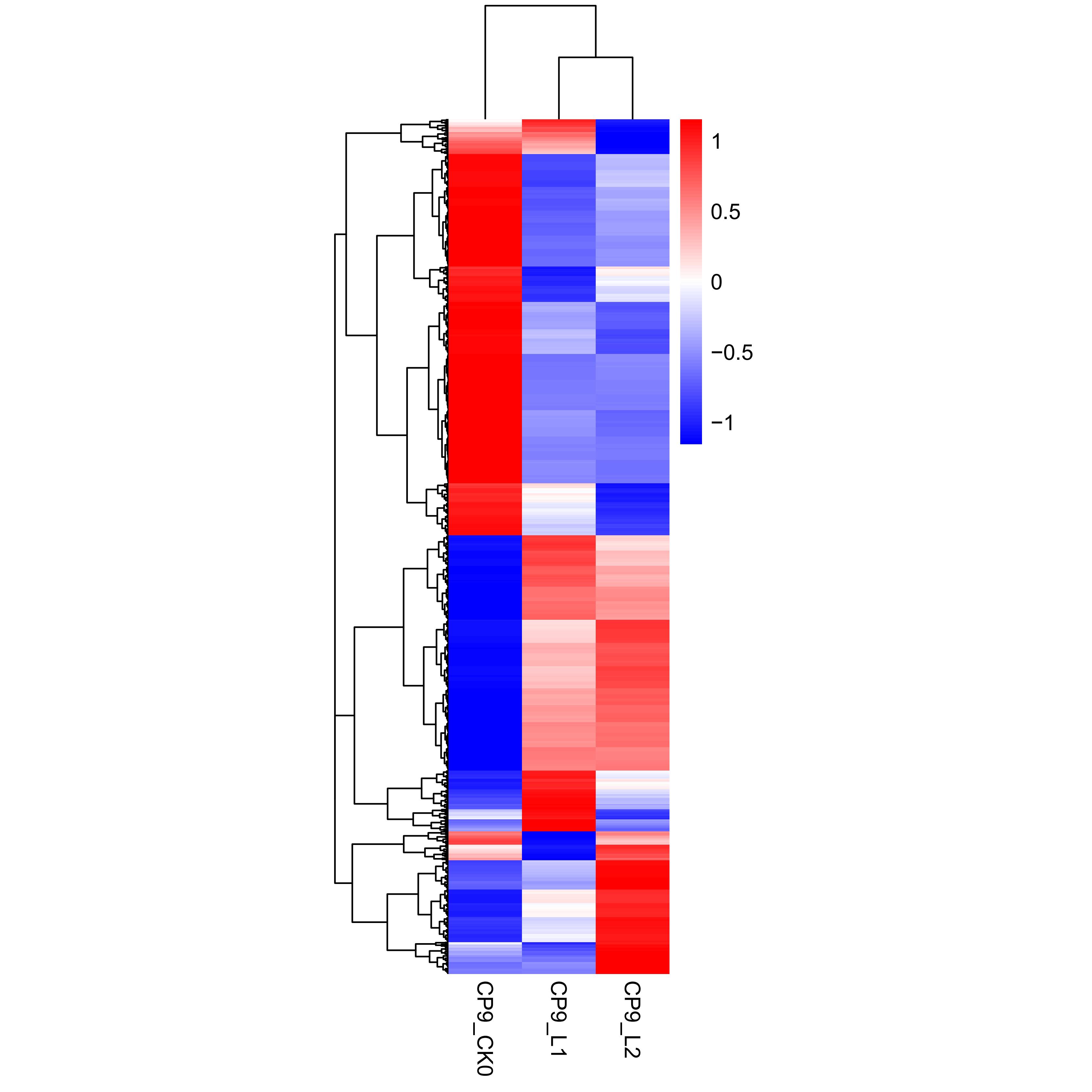

Supplement: Supplementary file 1 [file ijms-22-11308-s001.zip › Figure.S/Figure S1.jpg]

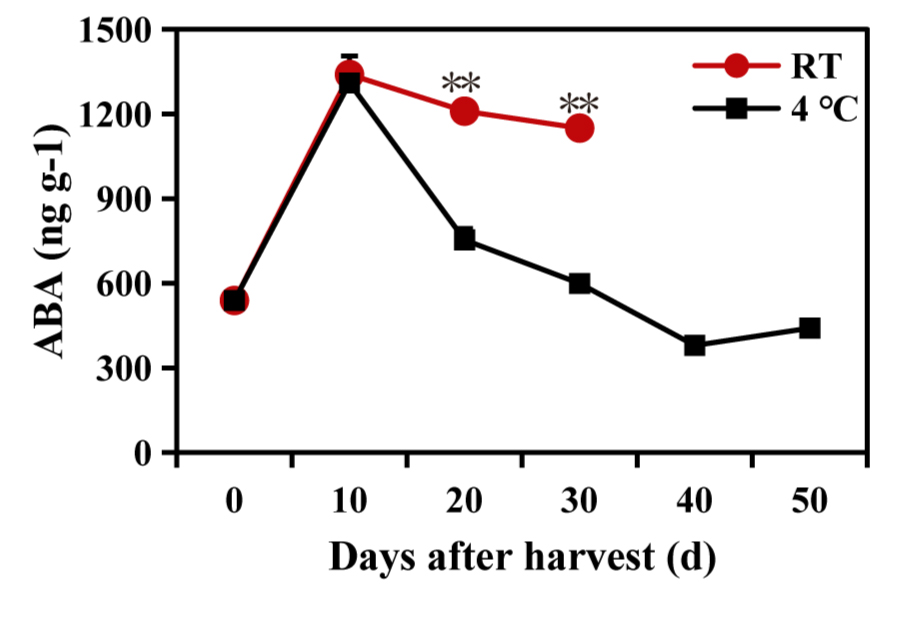

Supplement: Supplementary file 1 [file ijms-22-11308-s001.zip › Figure.S/Figure S2.jpg]

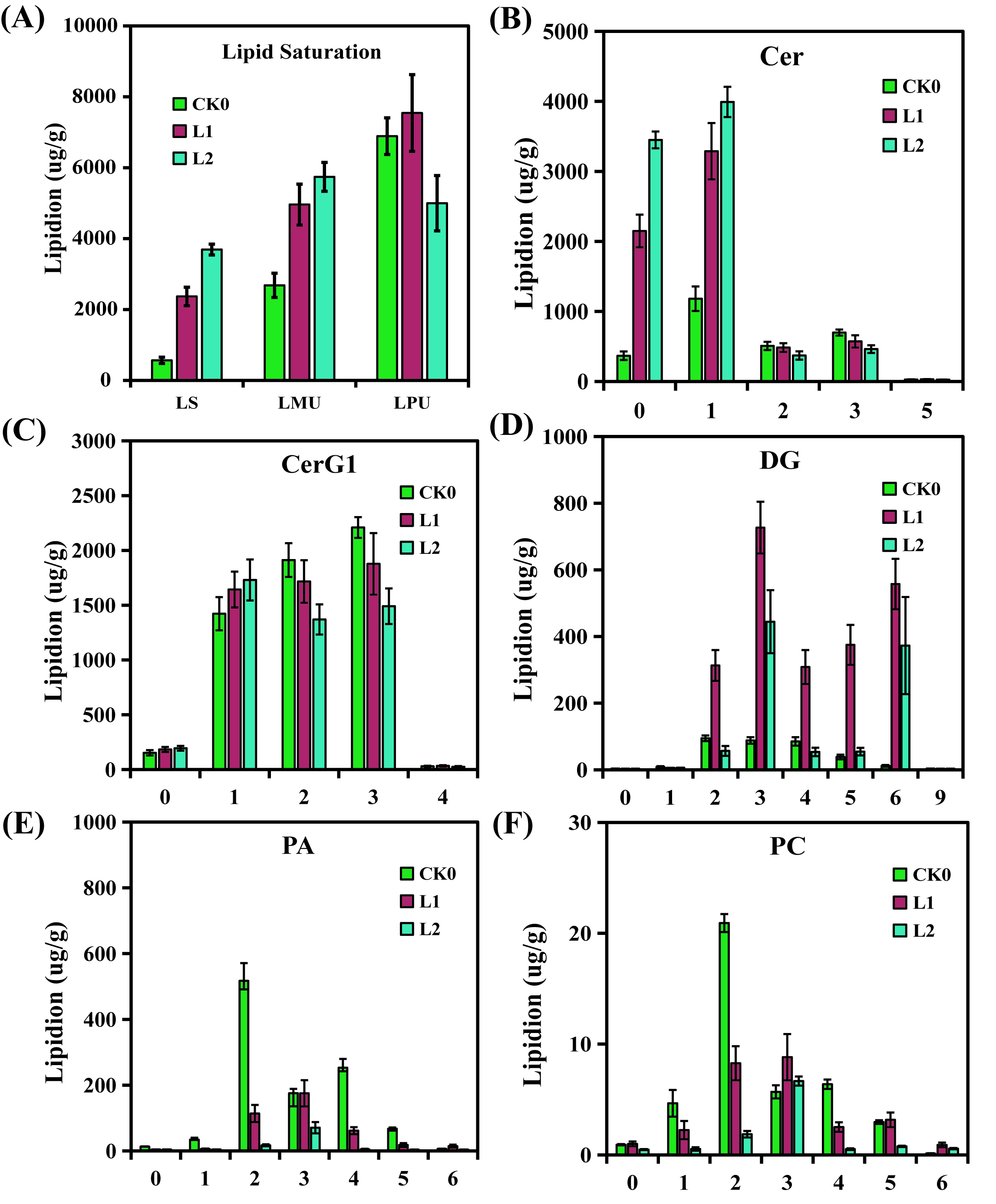

Supplement: Supplementary file 1 [file ijms-22-11308-s001.zip › Figure.S/Figure S3.jpg]

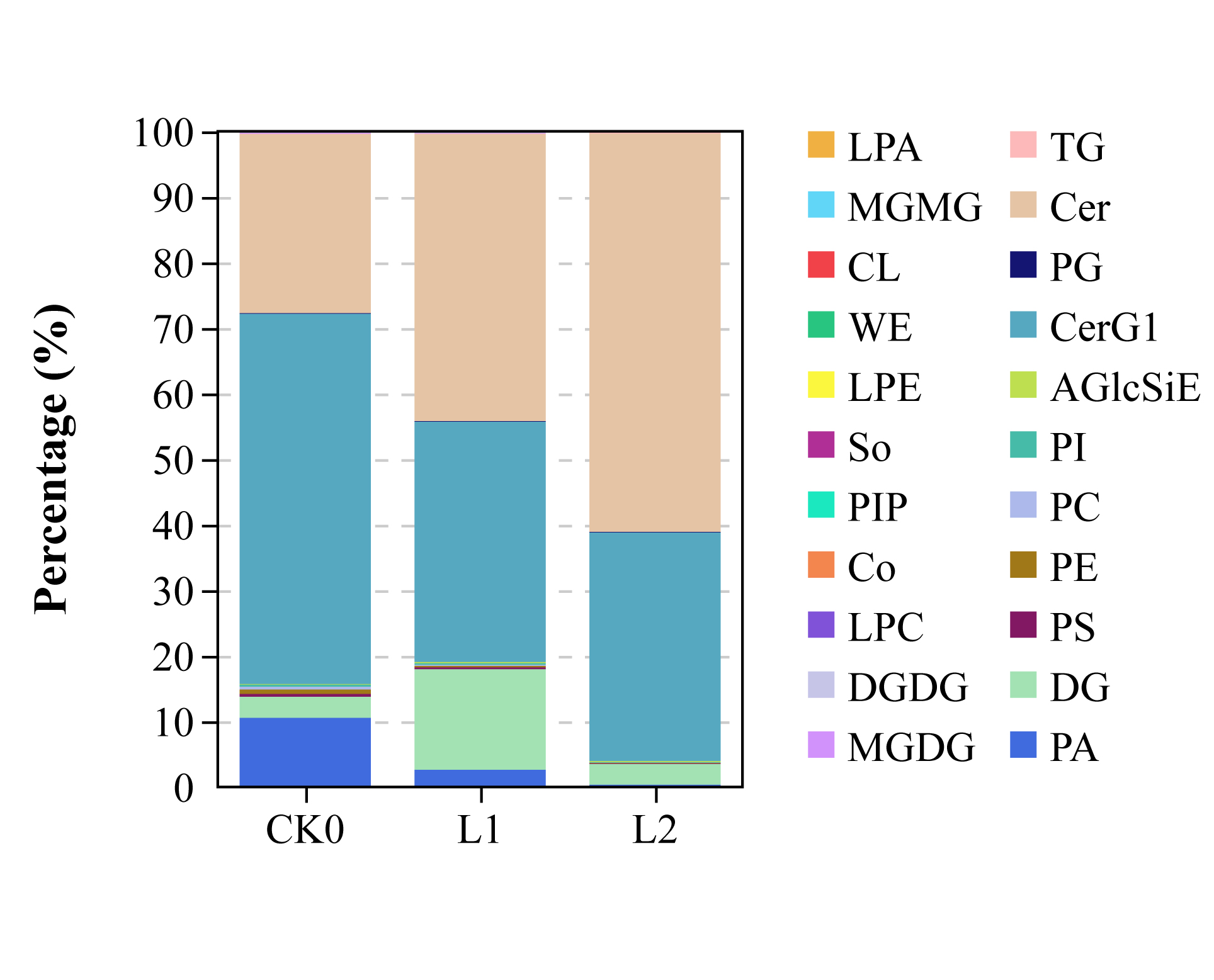

Supplement: Supplementary file 1 [file ijms-22-11308-s001.zip › Figure.S/Figure S4.jpg]

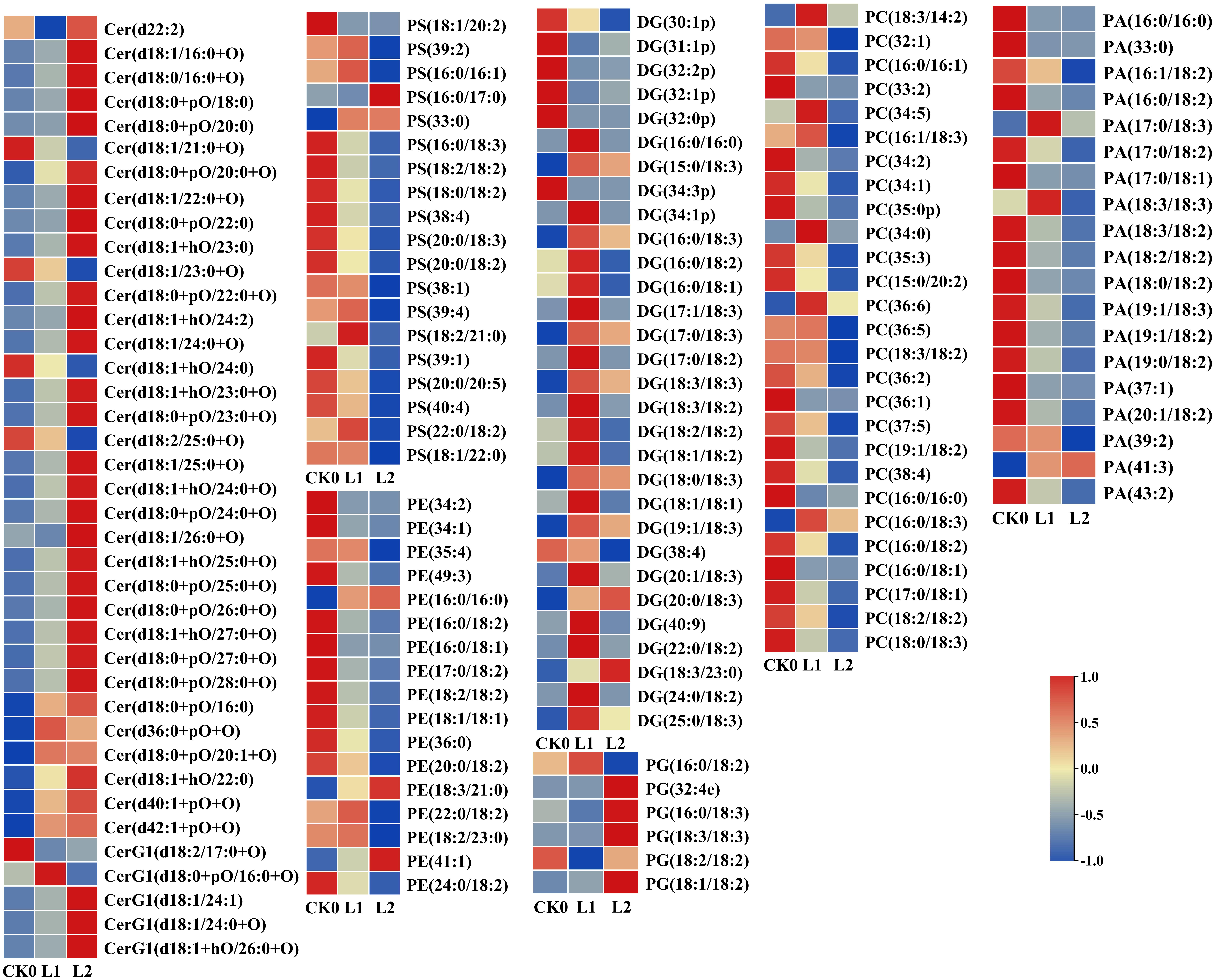

Supplement: Supplementary file 1 [file ijms-22-11308-s001.zip › Figure.S/Figure S5.jpg]

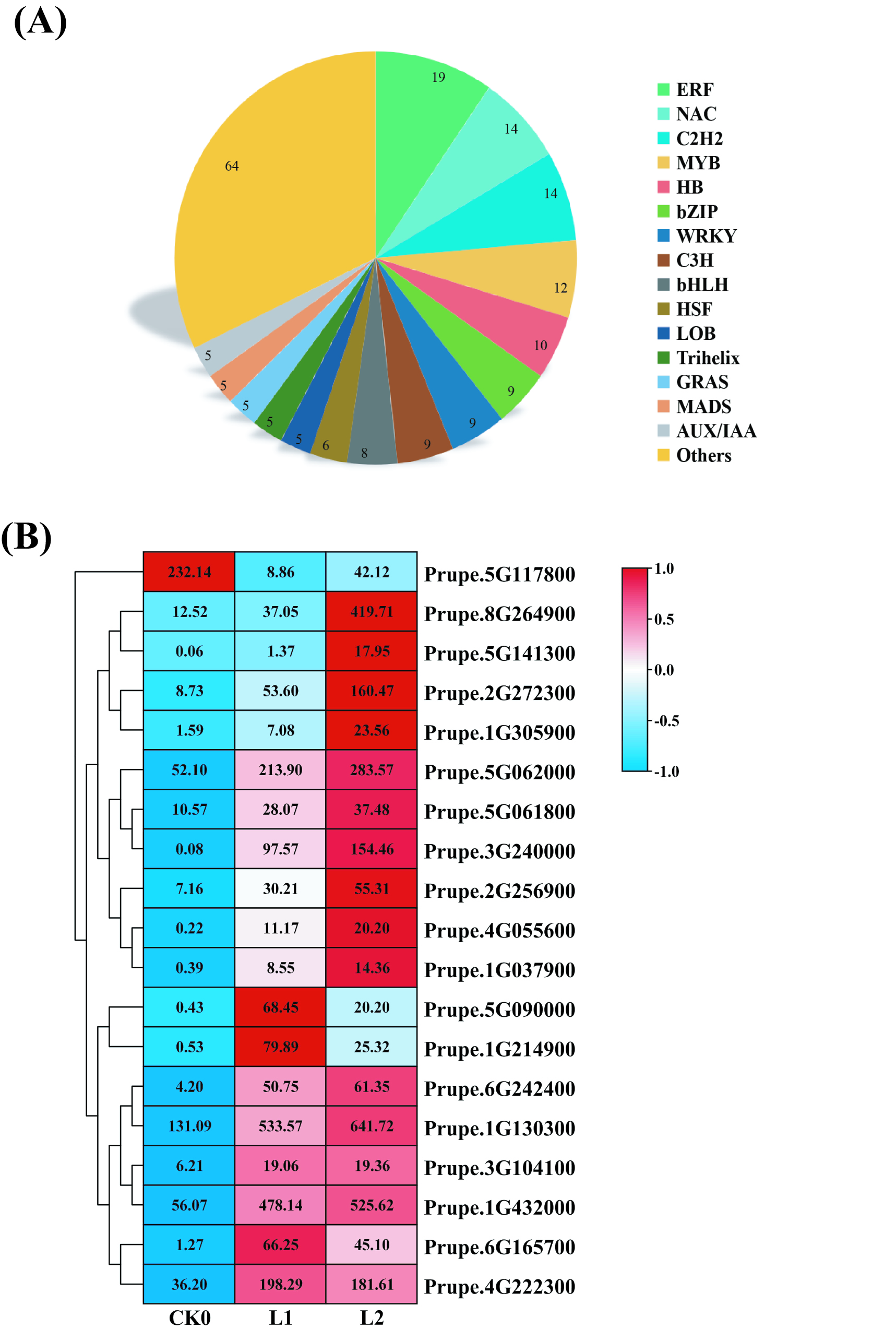

Supplement: Supplementary file 1 [file ijms-22-11308-s001.zip › Figure.S/Figure S6.jpg]
